# Perspectives of Canadian and American Cat Owners on Provision of Uncontrolled Outdoor Access for Owned Domestic Cats

**DOI:** 10.3389/fvets.2021.742245

**Published:** 2021-10-26

**Authors:** Sarah M. L. Tan, Sarah Jajou, Anastasia C. Stellato, Lee Niel

**Affiliations:** ^1^Faculty of Land and Food Systems, The University of British Columbia, Vancouver, BC, Canada; ^2^Department of Population Medicine, Ontario Veterinary College, University of Guelph, Guelph, ON, Canada

**Keywords:** *Felis catus*, outdoor access, perspectives, animal welfare, owner, cat

## Abstract

While uncontrolled outdoor access can increase opportunities for cat physical and mental stimulation, it can also increase risks of injury and illness, and result in predation of wild birds and small animals. In Canada and the United States, it is often recommended to keep cats indoors, but many owners still provide some level of outdoor access. The objectives of this study were to use a cross-sectional survey to explore the attitudes and practices of cat owners in Canada and the United States toward outdoor access and to identify factors that influence the provision of uncontrolled outdoor access. A convenience sample of cat owners (*N* = 7,838) were recruited to complete an online survey, and a mixed logistic regression model was used to examine associations between cat and owner-related factors, and uncontrolled outdoor access for cats, with province/state included as a random effect. In total, 57% of owners kept their cats indoors, and 43% provided some form of outdoor access, with 21% of total owners providing uncontrolled outdoor access. Provision of uncontrolled outdoor access was associated with factors related to cat characteristics (e.g., sex, breed, presence of health, and behavioral issues), the home environment (e.g., living with other pets, types of enrichment provided), owner perspectives on outdoor access (e.g., level of agreement with potential benefits and consequence of outdoor access), and owner demographics (e.g., gender, education, area of residence). For cats with uncontrolled outdoor access, few owners reported their cats having a collar or a microchip, suggesting a need to increase education about precautionary measures to protect the welfare of outdoor cats. Results reveal how owners are caring for their cats in terms of providing outdoor access and generate hypotheses for future research to examine the influence of the owner-pet bond and educational programs on owner practices around providing outdoor access.

## Introduction

It has been reported that 37% of Canadian households and 35% of American households own one or more cats (1, 2), which translates to ~9.3 million companion cats living within households in Canada (1) and 95.6 million in the United States (2). In recent years, both humane societies and wildlife organizations have developed educational campaigns to discourage cat owners from allowing uncontrolled outdoor access (i.e., free-roaming and unsupervised) due to the associated risks to both cats (3, 4) and wildlife (5, 6). Despite these educational campaigns, many cats are still allowed unrestricted outdoor access, without supervision. Based on a report from 2017, Canadian estimates suggest that 56% of owned domestic cats are housed indoors, 16% have outdoor access controlled (e.g., via direct supervision, enclosed area, and kept on a harness), and 28% are allowed at least some level of uncontrolled outdoor access (1).

It has been suggested that outdoor access has welfare benefits for cats since it promotes physical activity and natural behaviors (7), such as hunting, exploring, and climbing, and allows cats some level of autonomy to interact with their environment. Since cats were domesticated primarily for pest control, they remain highly motivated to perform predatory behaviors, including hunting and chasing (8), and have displayed preferences for climbing and perching on higher ground (7). Owned cats in England have also been reported to travel an average of 4.4 kilometers a day (9) when allowed outdoors. Indoor housing has been criticized as providing insufficient opportunities to meet these described needs. Cats confined to homes with limited space or opportunities for exercise are more likely to develop obesity, and other associated health issues (e.g., cardiorespiratory and urogenital disorders) (10). The inability to perform natural behaviors indoors has also been suggested to lead to frustration or boredom, resulting in the development of problematic behaviors (e.g., aggression, furniture scratching, or inappropriate elimination). Studies involving owner-completed surveys have found that some behavior problems are more prevalent in indoor cats than cats with outdoor access (11–14). However, another study found that behavior problems in indoor-restricted cats can be reduced through provision of some forms of enrichment (15).

In contrast, it has also been suggested that outdoor access has the potential to negatively impact cat health and welfare, as it exposes them to increased risks. For instance, cats that are allowed outdoors are at an increased risk of contracting diseases (e.g., feline immunodeficiency virus, rabies, and feline leukemia virus) or parasites through interaction with and exposure to other cats, and to wild or feral animals (8). Outdoor cats also have an increased risk of injury, predation, and poisoning. In areas with heavy traffic, particularly in urban cities, cats are at a higher risk of being involved in vehicle collisions which can cause serious injuries and acute or chronic health issues (7). Predation on cats by predators, such as dogs and coyotes, can also result in trauma and death of cats. Dog bites alone were reported to cause 10% (3/31) of cat trauma fatalities in a study at the Western College of Veterinary Medicine (16). Toxic hazards prevalent in neighborhood gardens or public parks, such as pesticide runoff, water contamination, and certain plants (e.g., lilies) can also increase risks of renal damage, vomiting, or death for outdoor cats (17).

Free-roaming cats can also negatively affect people and other animals. While freely roaming the outdoors, cats can be a nuisance to humans through excessive vocalization or inappropriate elimination on neighbors' properties (18, 19). They can also impact other animals through predation, with one study estimating that cat predation in the United States causes between 6.3 and 22.3 billion mammal deaths and between 1.3 and 4 billion bird deaths per year (20). While these numbers are staggering, most of this predation was attributed to unowned cats, with only 11% being attributed to owned, roaming cats. Further some have suggested these figures might be an overestimation of actual predation levels, and that there is little evidence of population-level impacts in terms of biodiversity as a result of cat predation (21). Regardless of debate over the overall impact of predation by owned cats, it is clear that outdoor access can, at a minimum, contribute to negative effects of cats on welfare and individual survival of wild species (22). Further, if owned cats do not return home they can contribute to unowned (stray or feral) cat population issues, and in turn, related predation. In 2016, Canadian shelters received an estimated 114,131 cats where 56% were admitted as strays and only 10% of these strays were returned to their owners (1).

Limited research has explored factors that influence owner decisions about provision of outdoor access in Canada and the US. In 2001, Clancy et al. (23) released a survey to 184 cat owners to assess owner attitudes toward outdoor access for cats in the US and found that 40% of cats had some degree of outdoor access; however, the authors did not differentiate between controlled and uncontrolled access. They found cats acquired from shelters were less likely to have outdoor access than cats acquired as strays, which they hypothesized was due to increased educational efforts from humane societies. They concluded that owners' decisions to provide outdoor access is multifactorial, suggesting a further study involving a broader population was necessary. In another large-scale study that was international in scope, significant regional differences were noted in attitudes and practices around provision of outdoor access, with owners in the UK, Europe, New Zealand, and Australia being more likely to provide uncontrolled outdoor access than owners in Canada and the US (24). Owners in this study that kept their cats indoors cited several reasons for indoor restriction including concerns about road traffic, protection from other people, animals or wildlife, protection of wildlife from predation, the cat getting lost, and factors related to the individual cat being unable to cope outdoors due to health or temperament. In contrast, reasons that were cited for allowing cats indoor-outdoor access included factors such as improving the cat's mental and physical health, pest control, having a multi-cat household, and the cat having had previous outdoor access.

While research in North America is limited, a number of studies from Australia and New Zealand have examined attitudes and practices of owners around cat containment. Recent figures from this region suggest that a majority of cat owners are allowing cats outdoors, with only 30 and 53% of study participants reporting containment at all times (25, 26). Studies from these regions have examined factors that influence intentions and actions around containment and highlighted various relevant factors. One study found that a majority of owners were concerned about cat safety and protection of native wildlife, with only half of participants being concerned about reducing unwanted breeding and preventing nuisance behavior (25). Further, support for containment at night in cat owners was related to stronger beliefs about impacts on wildlife and cat safety, and beliefs about containment predicted practices around containment. Other studies have also found that owner support for containment is associated with perceived benefits to that cat (26–28) and benefits to the owner (27), and some studies have also found that owner confidence around containment (28) and their perceived control is important (26). However, the influence of concerns about impacts on wildlife has been variable. While Toukhsati et al. (25) found that beliefs about wildlife were important to cat owners, other studies have found either no relationship or only a weak correlation (26, 27) between concerns about wildlife and containment perspectives. One recent study examined interventional messages about wildlife concerns and found that they were effective at increasing motivation to contain and belief that owners could contain (29), so it is possible that these differences between studies reflect a lack of understanding of the potential impact of cats on wildlife.

The overall objective of the current study was to better understand the attitudes and practices of Canadian and American cat owners toward provision of outdoor access through an online cross-sectional survey targeted to current cat owners. A number of specific factors relating to the cat, owner, and home environment have been hypothesized, based on previous literature as described above, to contribute to decisions about providing outdoor access, so a specific objective was to identity factors associated with the provision of uncontrolled outdoor access using regression modeling.

## Methods

This project was reviewed and approved by the University of Guelph (#18-08-019) and The University of British Columbia Research Ethics Boards (#H18-02597) for research involving human participants. A detailed electronic information letter was provided to participants at the landing page for the survey, and consent was demonstrated by participants submitting the survey responses following completion.

### Data Collection

Current cat owners completed an online cross-sectional survey that included questions about practices around provision of outdoor access, details of the home environment, and cat and owner demographic information. Inclusion criteria for participants required individuals be 18 years of age or older, a primary caregiver of at least one cat (e.g., routine financial and care responsibilities), and a current resident of either Canada or the United States of America. We used convenience sampling that involved recruitment via snowball sampling on social media, with the initial advertisement shared through Facebook and Twitter. This recruitment method relies on referrals from participants, as participants are encouraged to share the survey to recruit other persons who fit the specific criteria. Thus, from our initial posts, participants were asked to share the social media advertisement with their contacts. This sampling technique has been shown to efficiently reach targeted groups that are otherwise challenging to access (30, 31). The survey was advertised and available from October 31 to November 19, 2018.

### Questionnaire

The survey was created using Qualtrics® and was available online. The questionnaire was developed from current literature on topics related to acquisition sources (23), behavioral issues (32), outdoor access (8), and enrichment (15). The questionnaire was comprised of 43 questions categorized into four sections: cat characteristics (e.g., sex, age, breed, source, neuter status, health, and behavioral issues); home environment (e.g., living with other pets, type of outdoor access provided, enrichment techniques used); owner perspectives on outdoor access (e.g., level of agreement with potential benefits and consequences of outdoor access); and owner demographics (e.g., gender, education, age). Specific details about outdoor access were determined by asking yes-no closed-ended questions as to whether owners provide free-roaming unsupervised access, directly supervised access, enclosed outdoor access (e.g., catio), or access on a leash, harness or tie out. If participants owned more than one cat, they were instructed to respond to the survey for only one cat within the household. Selection bias was limited by asking them to respond based on the cat whose name begins with the letter closest to the beginning of the alphabet. In addition, participant responses were not connected to any directly or indirectly identifying information to minimize potential social desirability bias.

### Statistical Analysis

All analyses were performed with Stata Statistical Software v.15.1 (StataCorp, College Station, TX, USA).

#### Data Management

The initial dataset included 107 variables. During data cleaning, questions that had “other” as an option were cross-referenced with existing options to ensure answers were not misrepresented, thereby reducing misclassification bias. For ease of analysis, related variables were collapsed to create the following overarching variables: medical issues (e.g., gastrointestinal issues, skin conditions), aggression (e.g., toward people, cats), ownership of other non-cat pets (e.g., dogs, birds), and interactive enrichment (e.g., small toys, food devices).

#### Logistic Regression Model

A mixed logistic regression model was developed to test associations between independent variables and the dependent variable, owner-reported provision of uncontrolled outdoor access. Country and province/state were included as random effects. Referent categories for categorical variables were chosen based on biological plausibility or based on the most common response. Correlation analysis was performed on all retained variables, with a correlation coefficient of >|0.7| suggesting collinearity (33). Five correlations related to owner perspectives of outdoor access were detected during this assessment (perspective of access providing natural hunting behavior, natural environment, risk of obesity, natural exploratory behavior, and physical activity), and the most biologically meaningful variables that captured the most information were retained for further analysis (natural hunting behavior, natural environment, and physical activity) (33). Linear relationships between continuous independent variables and the outcome variable (uncontrolled outdoor access) were visually assessed using locally weighted regression curves (lowess) and quadratic relationships were assessed by testing the significance of a quadratic term. If the relationship was quadratic, the quadratic term was retained in the model. If the relationship was neither non-linear nor quadratic, the continuous variable was categorized. As a result of non-linear associations, the following variables were categorized based on biological/practical cut-points: participant age (18–24, 25–44, 45–64, 65+), time spent playing with the cats per day (<1, 1, 2, 3–12 h), and the number of cats owned (1, 2, 3+). Also, cat age was categorized based on the cat life stages presented in the AAFP 2010 guidelines (<4 months, 4–12 months, 1–6 years, 7–10 years, 11–14 years, and >15 years) (34).

Univariable analysis was performed to test each independent variable against the outcome, uncontrolled outdoor access. Variables were retained using a liberal *p*-value of *p* ≤ 0.20 (35). The final main effects model was built using forward stepwise selection method, where significant variables (*p* < 0.05) were retained in the final model. Two-way interactions between biologically plausible variables were tested. Confounders were tested based on their biological plausible relationship with an explanatory variable and the outcome. They were identified as a variable that caused >20% change in a coefficient of another variable in the model. Standardized Pearson residuals were used to detect outliers. The fit of the model was determined by assessing the homoscedasticity and normality of the best linear unbiased predictions (BLUPS). Also, the intra-class correlation coefficients (ICC) were estimated to measure the degree of correlation between cats owned within the same country and province/state.

## Results

### Descriptive Data

A total of 7,977 responses were collected from the survey and 7,838 complete responses were retained for analysis. The majority of participants resided in Canada (84.5% with 67% of participants residing in Ontario) and 15.5% of participants resided in the United States. Participants were 91.1% women, 5.9% men, and 3% who preferred not to disclose their gender identity or reported their identity was not listed. Participants had a mean age (SD) of 41.6 (13.8) years (range: 18–100+ years) and their corresponding owned cats had a mean age (SD) of 2.2 (1.9) years old (range: <4 months−20+ years). Cats were 50.5% female and 49.5% male, with 72.2% being domestic short-haired, 19.8% domestic long-haired, and 8% purebred.

**Figure 1:**
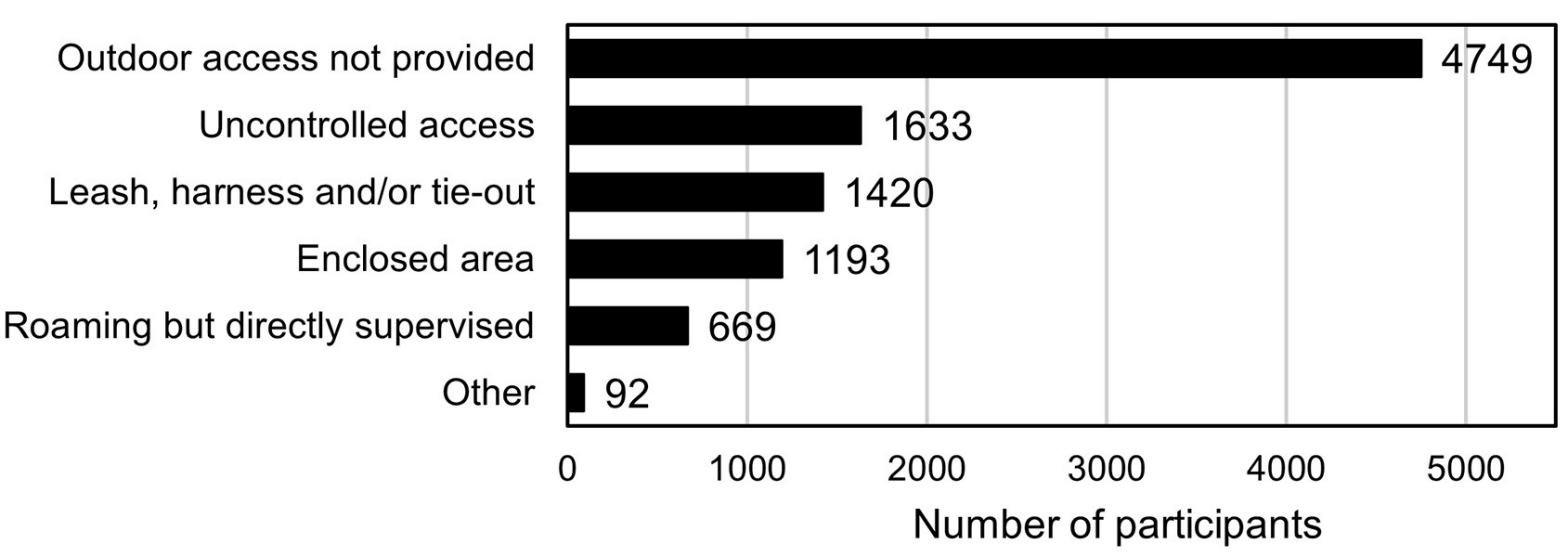
Owner-reported provision of outdoor access for companion cats, including method of provision (*N* = 7,838).

In total, 43% (*n* = 3,370) of owners provided some degree of outdoor access, with 21% of total owners allowing uncontrolled outdoor access and 22% providing controlled access via direct supervision, enclosed area, or being kept on a harness or leash (Figure 1). When owners were asked broadly about their agreement with provision of outdoor access for cats, 46.1% of owners agreed that cats with prior access to the outdoors should continue to be allowed outdoors, with 12.2% of owners unsure how prior access would impact their decision. Also, 78.3% of owners agreed that cats with no previous access should not be allowed outdoor access with 7.4% of owners unsure how no previous access would influence their decision of providing access.

Cat management factors that have the potential to influence cat welfare when cats are allowed outside or kept solely indoors are summarized and presented in Tables 1, 2. Fewer cats with uncontrolled outdoor access were declawed and more were provided with collars or belled collars compared to cats without uncontrolled outdoor access (controlled or indoor). Indoor cats had more access to enrichment, such as interactive toys, elevated platforms, scratching areas, and exploratory items. Similar percentages were detected in regard to being licensed (21.8%, 25.7%) and neutered (97.2%, 97.9%) between cats with uncontrolled and controlled outdoor access.

**Table 1:** Summary statistics for owner-reported provision of enrichment opportunities for cats with (*n* = 1,633) and without (*n* = 4,896) uncontrolled outdoor access.

		**Uncontrolled outdoor access**	**No uncontrolled outdoor access**
Active play time	<1 h	548 (33.7%)	1,366 (28.0%)
	1 h	527 (32.4%)	1,612 (33.0%)
	2 h	324 (19.9%)	1,066 (21.8%)
	3–12 h	229 (14.1%)	843 (17.2%)
Small toys (e.g., furry mice, crinkle sacks)	Yes	1,447 (88.8%)	4,770 (97.5%)
	No	182 (11.2%)	120 (2.5%)
Interactive toys (e.g., feather wand)	Yes	1,115 (69.9%)	4,190 (85.9%)
	No	479 (30.1%)	690 (14.1%)
Feeding device (e.g., puzzle feeders)	Yes	303 (19.4%)	1,349 (27.8%)
	No	1,258 (80.6%)	3,504 (66.0%)
Elevated platforms (e.g., perches)	Yes	1,241 (76.9%)	4,389 (89.9%)
	No	373 (23.1%)	492 (10.1%)
Scratching areas (e.g., scratching post)	Yes	1,311 (81.3%)	4,506 (92.2%)
	No	302 (18.7%)	383 (7.8%)
Exploratory items (e.g., tunnels, boxes)	Yes	1,034 (64.9%)	3,848 (78.9%)
	No	558 (35.1%)	1,031 (21.1%)
Olfactory stimulation (e.g., catnip)	Yes	860 (53.9%)	3,169 (65.0%)
	No	736 (46.1%)	1,703 (35.0%)
Training (e.g., clicker training)	Yes	154 (9.4%)	677 (13.8%)
	No	1,477 (90.6%)	4,213 (86.2%)
Outdoor access under direct supervision	Yes	112 (12.8%)	368 (7.5%)
	No	766 (87.2%)	4,507 (92.5%)
Enclosed outdoor access (e.g., catio, arched cat fencing)	Yes	42 (4.9%)	743 (15.3%)
	No	823 (95.1%)	4,118 (84.7%)
Outdoor access via leash, harness and/or tie out	Yes	27 (3.1%)	1,007 (20.8%)
	No	840 (96.9%)	3,840 (79.2%)

*Cats with uncontrolled outdoor access were allowed opportunities to freeroam while unsupervised. Cats with no uncontrolled outdoor access included both indoor-only cats, and cats that were allowed outdoors in a controlled manner (e.g., under direct supervision, kept in an enclosed area, or on a harness or leash)*.

**Table 2:** Summary statistics for owner-reported characteristics of cat management that have the potential to impact the welfare of cats during outdoor access for cats with (*n* = 1,633) and without (*n* = 4,896) uncontrolled outdoor access.

**Cat characteristics**		**Uncontrolled outdoor access**	**No uncontrolled outdoor access**
Declaw status	Yes	110 (6.7%)	612 (12.5%)
	No	1,523 (93.3%)	4,282 (87.5%)
Microchip status	Yes	676 (41.5%)	2,632 (53.8%)
	No	910 (55.8%)	2,129 (43.5%)
	Unsure	44 (2.7%)	135 (2.8%)
Collar	Yes	445 (27.3%)	581 (11.9%)
	No	1,167 (71.7%)	484 (9.9%)
	Indoor only	16 (1.0%)	3,811 (73.2%)
Collar with bell	Yes	261 (16.0%)	418 (8.6%)
	No	654 (40.2%)	531 (10.9%)
	Indoor only	13 (0.8%)	3,103 (63.5%)
	No collar	701 (43.0%)	837 (17.1%)
License	Yes	353 (21.8%)	1,258 (25.7%)
	No	1,124 (68.9%)	3,111 (63.6%)
	Unsure	151 (9.3%)	521 (10.7%)
Neuter status	Yes	1,598 (97.2%)	4,758 (97.9%)
	No	31 (2.7%)	133 (1.9%)
	Unsure	4 (0.1%)	3 (0.2%)

*Cats with uncontrolled outdoor access were allowed opportunities to freeroam while unsupervised. Cats with no uncontrolled outdoor access included both indoor-only cats, and cats that were allowed outdoors in a controlled manner (e.g., under direct supervision, kept in an enclosed area, or on a harness or leash)*.

### Risk Factors

Risk factors of uncontrolled outdoor access are presented in Tables 3–**7**, and the final model included factors related to cat characteristics, the home environment, owner perspectives on outdoor access, and owner demographics.

**Table 3:** Logistic regression model for risk factors associated with uncontrolled outdoor access for cats based on owner reports, with province/state asLiving location a random effect (*N* = 7,838 participants).

**Risk factors**		**OR^**a**^**	**95% CI^**b**^**	***P***
Sex		-	-	<0.001
	Female (ref.)^c^	-	-	-
	Male	1.58	1.28, 1.94	**<0.001**
Breed		-	-	<0.001
	Domestic short-haired cat (ref.)	-	-	-
	Domestic long-haired cat	1.03	0.75, 1.26	0.832
	Purebred cat	0.30	0.16, 0.56	**<0.001**
Age		-	-	<0.001
	1–6 years (ref.)	-	-	-
	<4 months	0.02	0.001, 0.29	**0.005**
	4–12 months	0.24	0.13, 0.45	**<0.001**
	7–10 years	1.24	0.95, 1.61	0.116
	11–14 years	1.36	0.99, 1.88	0.060
	15+ years	2.33	1.54, 3.52	**<0.001**
Medical condition		-	-	0.003
	No (ref.)	-	-	-
	Yes	0.68	0.53, 0.88	**0.003**
Aggression		-	-	<0.001
	No (ref.)	-	-	-
	No opportunity to assess	0.38	0.09, 1.68	0.203
	Yes	1.70	1.38, 2.10	**<0.001**
Scratch		-	-	0.0068
	No (ref.)	-	-	**-**
	No opportunity to assess	5.06	1.84, 13.90	**0.002**
	Yes	1.01	0.82, 1.25	0.924
Indoor contract acquisition source		-	-	<0.001
	No (ref.)	-	-	-
	Unsure	0.59	0.39, 0.88	**0.010**
	Yes	0.43	0.29, 0.63	**<0.001**
		-	-	0.003
	Cat rescue or shelter (ref.)	-	-	-
	Cattery	0.69	0.24, 2.03	0.504
	Classified advertisement	1.05	0.70, 1.56	0.825
	Family or friend	1.21	0.89, 1.65	0.224
	Free-roaming stray	1.88	1.39, 2.56	**<0.001**
	Pet store	1.36	0.86, 2.14	0.187
	Other	0.83	0.41, 1.70	0.614

*Cat characteristics reported here, with other aspects of the model reported in Tables 4–7*.

a *Odds ratio based on the output of mixed logistic regression model*.

b *95% confidence interval of the odds ratio*.

c *Referent category*.

*Bolded values indicate significance (p < 0.05)*.

#### Cat Characteristic Factors

Male cats and cats older than 15 years of age had higher odds of being allowed uncontrolled outdoor access (Table 3). Cats who had aggressive behavioral issues directed toward people or other animals in and out of the household, also had increased odds of uncontrolled outdoor access. In contrast, cats who were less than a year old, were purebred, or had an existing medical condition, had decreased odds of being provided with uncontrolled outdoor access. If the owner reported that they had signed an indoor contract upon acquiring the cat, there was lower odds of the owner providing uncontrolled outdoor access.

#### Owner Demographic Factors

Owners who lived in a village (with a population of less than a thousand people) or resided on a farm had significantly higher odds of allowing their cat uncontrolled outdoor access (Table 4). In contrast, owners living in an apartment, condominium, townhouse, or semi-detached house had lower odds of giving their cats uncontrolled outdoor access. Women and owners who had a professional degree (e.g., veterinarian) also had lower odds of providing uncontrolled outdoor access to their cats.

**Table 4:** Logistic regression model for risk factors associated with uncontrolled outdoor access for cats based on owner reports, with province/state as a random effect (*N* = 7,838 participants).

**Risk factors**		**OR^**a**^**	**95% CI^**b**^**	***P***
Gender		-	-	0.013
	Man (ref.)^c^	-	-	-
	Woman	1.64	1.03, 2.60	**0.037**
	My gender identity is not listed above	4.02	1.41, 11.50	**0.009**
	Prefer not to answer	3.23	1.29, 8.13	**0.013**
Education		-	-	0.007
	College Certificate or Diploma (ref.)	-	-	-
	Elementary school	0.25	0.04, 1.56	0.138
	Secondary school	1.26	0.91, 1.75	0.170
	Bachelor's degree	0.78	0.60, 1.02	0.068
	Master's degree	0.68	0.46, 1.00	0.050
	Doctor of Philosophy (Ph.D.) degree	0.67	0.34, 1.33	0.250
	Professional degree	0.49	0.30, 0.78	**0.003**
	Prefer not to answer	0.61	0.29, 1.29	0.198
Living location		-	-	<0.001
	Large city (300,000–1 million people) (ref.)	-	-	-
	Village (<1,000 people)	1.85	1.22, 2.79	**0.003**
	Small town (1,000–20,000 people)	1.30	0.92, 1.82	0.134
	Large town (20,000–100,000 people)	0.97	0.68, 1.38	0.858
	Small city (100,000–300,000 people)	0.76	0.54, 1.07	0.116
	Metropolis (>1 million people)	1.19	0.74, 1.91	0.471
Household type		-	-	<0.001
	Detached house (ref.)	-	-	-
	Apartment/condo	0.15	0.10, 0.24	**<0.001**
	Townhouse/semi-detached	0.60	0.43, 0.83	**0.002**
	Trailer home	1.43	0.55, 3.73	0.466
	Farm/acreage	6.92	3.17, 15.11	**<0.001**
	Other	1.48	0.47, 4.62	0.505
	Prefer not to answer	0.69	0.13, 3.60	0.662

*Owner demographics reported here, with other aspects of the model reported in Tables 3, 5–7*.

a *Odds ratio based on the output of mixed logistic regression model*.

b *95% confidence interval of the odds ratio*.

c *Referent category*.

*Bolded values indicate significance (p < 0.05)*.

#### Owner Perspectives

**Figure 2:**
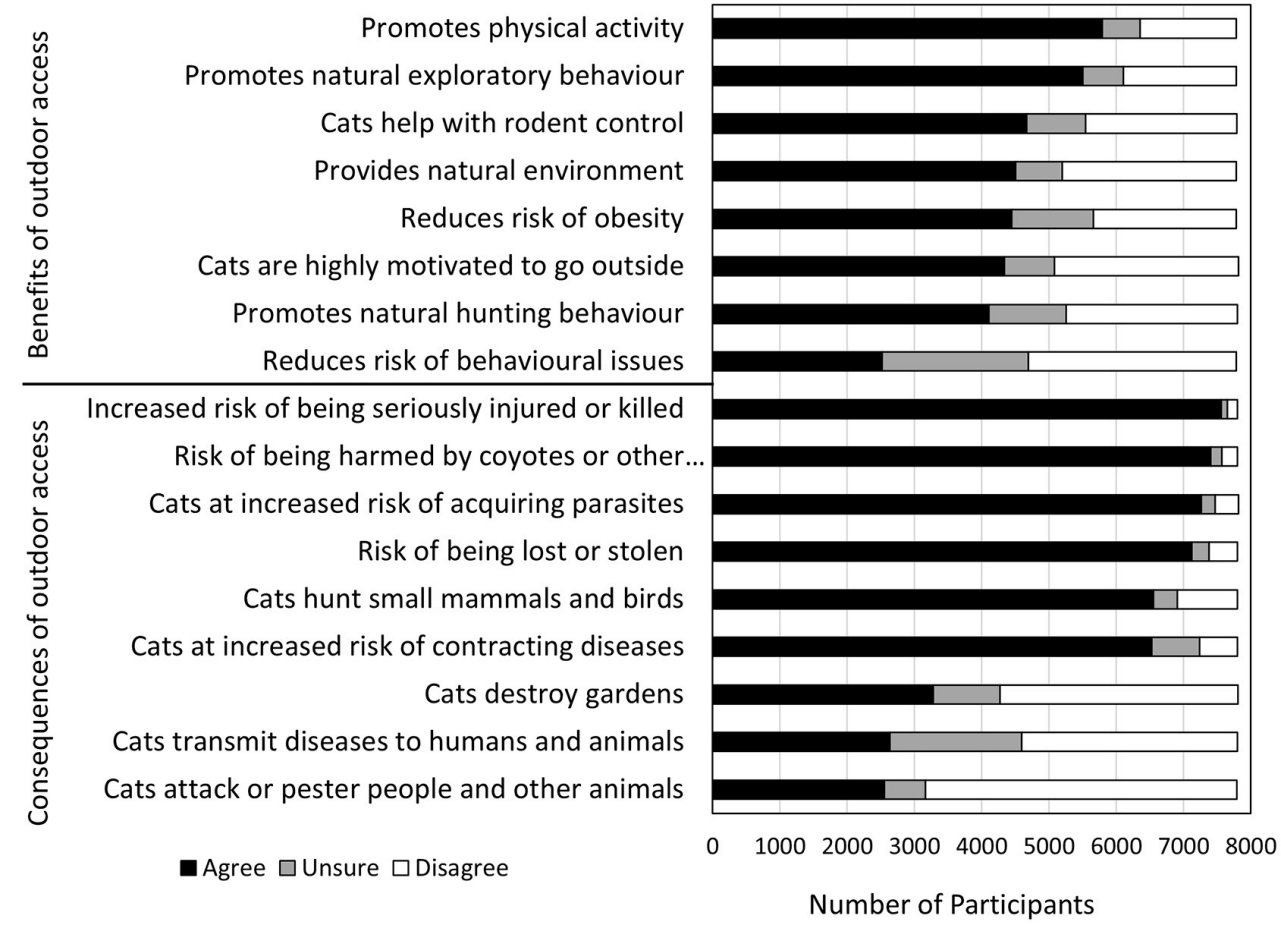
Owner responses on level of agreement with statements on the benefits and consequences of outdoor access for companion cats (*N* = 7,838).

Owners showed a range of perspectives on the benefits and risks associated with outdoor access (Figure 2). In general, owners who agreed with risks associated with outdoor access (e.g., contracting a disease) had lower odds of allowing uncontrolled outdoor access, and those who agreed with benefits of providing outdoor access (e.g., increased activity) had higher odds of letting their cats outside uncontrolled (Tables 5, 6).

**Table 5:** Logistic regression model for risk factors associated with uncontrolled outdoor access for cats based on owner reports, with province/state as a random effect (*N* = 7,838 participants).

**Risk factors**		**OR^**a**^**	**95% CI^**b**^**	***P***
Cats are highly motivated to go outside		-	-	<0.001
	Somewhat agree (ref.)^c^	-	-	-
	Strongly agree	1.65	1.27, 2.13	**<0.001**
	Somewhat disagree	0.40	0.27, 0.60	**<0.001**
	Strongly disagree	0.17	0.08, 0.38	**<0.001**
	Unsure	0.46	0.30, 0.70	**<0.001**
Cats can help with rodent control		-	-	0.0046
	Somewhat agree (ref.)	-	-	-
	Strongly agree	1.01	0.78, 1.31	0.952
	Somewhat disagree	0.92	0.64, 1.33	0.670
	Strongly disagree	0.411	0.24, 0.71	**0.001**
	Unsure	0.66	0.46, 0.94	0.022
Promotes natural hunting behavior		-	-	0.0029
	Somewhat agree (ref.)	-	-	-
	Strongly agree	0.83	0.62, 1.12	0.188
	Somewhat disagree	1.72	1.20, 2.47	**0.0030**
	Strongly disagree	1.69	0.99, 2.88	0.054
	Unsure	1.33	0.97, 1.81	0.075
Reduces risk of behavioral issues		-	-	<0.001
	Somewhat agree (ref.)	-	-	-
	Strongly agree	1.53	1.11, 2.11	**0.010**
	Somewhat disagree	0.62	0.43, 0.90	**0.013**
	Strongly disagree	0.69	0.41, 1.18	0.1810
	Unsure	0.72	0.55, 0.95	**0.018**
Promotes physical activity		-	-	<0.001
	Somewhat agree (ref.)	-	-	-
	Strongly agree	1.36	1.05, 1.76	**0.018**
	Somewhat disagree	0.31	0.16, 0.63	**0.0010**
	Strongly disagree	0.15	0.03, 0.72	**0.018**
	Unsure	0.64	0.37, 1.09	0.1000

*Owner perspectives on the benefits of outdoor access reported here, with other aspects of the model reported in Tables 3, 4, 6, 7*.

a *Odds ratio based on the output of mixed logistic regression model*.

b *95% confidence interval of the odds ratio*.

c *Referent category*.

*Bolded values indicate significance (p < 0.05)*.

**Table 6:** Logistic regression model for risk factors associated with uncontrolled outdoor access for cats based on owner reports, with province/state as a random effect (*N* = 7,838 participants).

**Risk factors**		**OR^**a**^**	**95% CI^**b**^**	***P***
Increases risk of contracting diseases		-	-	<0.001
	Somewhat agree (ref.)^c^	-	-	-
	Strongly agree	0.49	0.38, 0.64	**<0.001**
	Somewhat disagree	1.31	0.85, 2.02	0.221
	Strongly disagree	1.13	0.60, 2.13	0.714
	Unsure	1.05	0.76, 1.45	0.758
Cats hunt small mammals and birds		-	-	<0.001
	Somewhat agree (ref.)	-	-	-
	Strongly agree	1.75	1.37, 2.25	**<0.001**
	Somewhat disagree	0.99	0.70, 1.41	0.960
	Strongly disagree	1.53	0.88, 2.67	0.133
	Unsure	0.98	0.58, 1.67	0.948
Increased risk of injury or death		-	-	<0.001
	Somewhat agree (ref.)	-	-	-
	Strongly agree	0.49	0.36, 0.67	**<0.001**
	Somewhat disagree	0.64	0.29, 1.44	0.281
	Strongly disagree	0.55	0.13, 2.32	0.411
	Unsure	0.49	0.20, 1.17	0.109
Increased risk of being lost or stolen		-	-	0.0125
	Somewhat agree (ref.)	-	-	-
	Strongly agree	0.65	0.49, 0.85	**0.0020**
	Somewhat disagree	1.04	0.64, 1.69	0.864
	Strongly disagree	1.53	0.57, 4.07	0.399
	Unsure	1.23	0.72, 2.13	0.443
Increased risk of being harmed by coyotes or other wildlife		-	-	<0.001
	Somewhat agree (ref.)	-	-	-
	Strongly agree	0.50	0.38, 0.67	**<0.001**
	Somewhat disagree	1.23	0.69, 2.20	0.480
	Strongly disagree	1.21	0.26, 5.51	0.809
	Unsure	1.25	0.67, 2.36	0.486
Cats with prior outdoor access should continue to have outdoor access		-	-	<0.001
	Somewhat agree (ref.)	-	-	-
	Strongly agree	2.43	1.86, 3.17	**<0.001**
	Somewhat disagree	0.45	0.32, 0.63	**<0.001**
	Strongly disagree	0.15	0.07, 0.29	**<0.001**
	Unsure	0.76	0.44, 1.07	0.118

*Owner perspectives on the consequences of outdoor access reported here, with other aspects of the model reported in Tables 3–5, 7*.

a *Odds ratio based on the output of mixed logistic regression model*.

b *95% confidence interval of the odds ratio*.

c *Referent category*.

*Bolded values indicate significance (p < 0.05)*.

#### Home Environment and Enrichment Factors

Cat owners who had additional pets (e.g., dogs, fish, reptiles, and birds) in the home, had higher odds of providing uncontrolled outdoor access (Table 7). However, if owners provided interactive enrichment, such as feather wands, small toys, exploratory devices, and elevated platforms, the odds of the owner allowing uncontrolled outdoor access were lower.

**Table 7:** Linear regression model for risk factors associated with uncontrolled outdoor access for cats based on owner reports, with province/state as a random effect (*N* = 7,838 participants).

**Risk factors**		**OR^**a**^**	**95% CI^**b**^**	***P***
**Home Environment and Enrichment**				
Other pets (non-cats)		-	-	<0.001
	No (ref.)^c^	-	-	-
	Yes	2.14	1.69, 2.70	**<0.001**
Interactive toys		-	-	<0.001
	No (ref.)	-	-	-
	Yes	0.31	0.17, 0.56	**<0.001**
Elevated platforms		-	-	<0.001
	No (ref.)	-	-	-
	Yes	0.61	0.46, 0.81	**0.0010**
**Random effect**				
Province/State	ICC	0.055	0.017, 0.17	<0.0001
	Province/State-level variance	0.19	0.006, 0.67	-

*Home environment and enrichment factors plus random effects reported here, with other aspects of the model reported in Tables 3–6*.

a *Odds ratio based on the output of mixed logistic regression model*.

b *95% confidence interval of the odds ratio*.

c *Referent category*.

*Bolded values indicate significance (p < 0.05)*.

#### Interactions

Significant interactions were found between the: cat's acquisition source and owner's agreement that outdoor access is beneficial for rodent control; cat's age and owner's agreement that cats are highly motivated to go outside; and owner's gender and agreement that outdoor access promotes physical activity. After reviewing the tested interactions, it was determined that the significant interactions involving owner perspectives were not meaningful. As a result, the interactions were not included in the final model, allowing for a more intuitive and parsimonious model. No biologically plausible confounders were identified for further assessment. Country was not a significant random effect and was thus excluded. The random effect, province/state, was significant, and based on the ICCs (95% CI) of the model, cats within the same province/state have a correlation of 0.055 (0.017, 0.17) (Table 7).

## Discussion

Despite recent educational messages from animal welfare and conservation organizations in Canada and the US, results from the current study suggest that a large proportion of cat owners are providing uncontrolled outdoor access to their cats. While the majority of owners believed that cats with no prior outdoor experience should be kept indoors, there were still 1,119 owners (14.3%) who disagreed. This suggests that outdoor access does not depend on prior access experience as close to 21% of owners would still offer access to a new, inexperienced cat. There are also many owners allowing controlled outdoor access by having their cat on a leash, harness or tie-out, offering a catio or an enclosed area with cat-specific fencing, or letting the cats roam but only under direct supervision.

For cats who were allowed uncontrolled outdoor access, the majority of owners neutered their animals, which is important for reducing cat overpopulation and preventing hormonally mediated roaming. While 93% of outdoor cats had their claws, 7% of the uncontrolled outdoor cats were declawed, which is concerning as these animals are likely to be unable to defend themselves against predation. Few owners in this sample took measures to reduce potential cat loss through identification via microchip or collar or through licensing. One previous study found that a majority of cat owners agreed that microchipping helps lost cats reunite with their owners, but did not agree with cat licensing (19). This study did not assess the actual practices of these owners around microchipping and licensing, but another recent study from Australia found that 72% of cat owners reported microchipping their cat, which is much higher than what was found for the current study (36). Permanent identification (e.g., via microchip or tattoo) or provision of identification on a collar are commonly recommended to ensure the cat and owner can be identified and reunited if the cat is injured or becomes lost while outdoors (37).

### Cat Characteristic Factors

The current results suggest that male cats are more likely to be provided uncontrolled outdoor access than female cats, which corresponds to the findings of another recent study with international scope (24). In a previous US study on outdoor access, access did not differ between female and male cats (23), but the sample size in that study was relatively small. It has been suggested that female cats might be more suited to live solely indoors compared to male cats based on differences in home range sizes. Mertens and Schär (38) observed that indoor, neutered males had a home range of 4–5 rooms, compared to 3–3.6 rooms for neutered females. As a result, male cats might be more motivated to roam than females, potentially leading to frustration and behavioral issues when restricted. However, the home range differences are relatively small and differences in motivation to roam have not been assessed in neutered male and female cats. Alternatively, owners might be more worried about female cats having uncontrolled outdoor access because of the potential for pregnancy. However, in the current study, 97% of the cats were neutered or spayed, so this hypothesis is unlikely to be a primary contributor to the provisioning of outdoor access.

Cat aggression toward people or other animals was also a factor associated with increased likelihood of outdoor access. However, the directionality of the relationship between aggression and outdoor access is unknown; cats might be let outside because of aggressive behavior, or cats might become aggressive due to the outdoor access provided. Levine et al. (39) observed that households with at least one cat with outdoor access experienced more inter-cat aggression than when all cats were kept indoors. They hypothesized that either cats with outdoor access bring new smells into the home creating aggression in the indoor cat(s), or inter-cat aggression results from redirected frustration from not receiving outdoor access (39).

In addition, cats older than 15 years had higher odds of outdoor access and cats younger than a year of age had lower odds. Foreman-Worsley et al. (24) also found that juvenile cats under 2 years of age were more likely to be kept indoors. It is possible that older cats have a higher probability of having previous outdoor access based on recently updated recommendations for keeping cats indoors, as owners reported strong agreement with continuing to provide outdoor access for cats with prior experience. In contrast, younger cats may be kept inside because they are not large enough to protect themselves or because they are not fully vaccinated or spayed/neutered [procedures typically done before 6 months of age (40)]; unvaccinated cats, particularly those younger than 4 months of age, would be more susceptible to contracting diseases, parasites, or illness, and unneutered young cats that are past sexual maturity would be capable of reproduction.

Similar to the results of Foreman-Worsley et al. (24), the current study found that cats who have an existing medical problem were less likely to have uncontrolled outdoor access. Cats with existing medical issues might be more susceptible to acquiring a disease or infection and/or vulnerability to predation. For example, particular diseases that are immunosuppressive and increase the risk of contracting secondary infections (41), such as Feline Leukemia Virus, might result in owners being less likely to allow their cat outdoors. Additionally, some medical issues (e.g., cancer and diabetes) require supportive care, such as assisted feeding and scheduled medication delivery (42), which could deter owners from allowing uncontrolled outdoor access as returning times could be unpredictable.

At many humane societies and animal shelters, adopters are required to sign contracts that ensure cats remain solely indoors, and our results suggest these contracts are effective at reducing the provision of uncontrolled outdoor access. It is possible that owners who acquired their cats from shelters are more educated about the potential consequences associated with outdoor access through educational materials provided by the shelter or breeder, and thus may be less likely to provide uncontrolled access. A similar relationship was discussed by Clancy et al. (23), who found a significant difference in provision of outdoor access between cats that were initially acquired from a shelter vs. as a stray, suggesting the prevention of uncontrolled outdoor access was due to the education provided by the shelters. However, some owners who had signed contracts were still providing uncontrolled outdoor access, suggesting that these contracts are not fully effective.

### Owner Demographic Factors

The types of dwellings and areas that cat owners reside in influenced whether cats are allowed outside, with more uncontrolled outdoor access provided for cats in rural areas in comparison to urban areas; these results correspond with another study that found indoor restriction is associated with city centers and urban areas (24). Owners in urban areas have a greater likelihood of living in an apartment or housing with multiple floors that lack direct outdoor access compared to living in rural areas. Higher outdoor access in rural and farm areas might also relate to cats being kept as domestic predators to control pests (43), as confirmed by some participants in this study, who specifically stated in their comments that they owned cats for this purpose. Also, free-roaming cats have a higher likelihood of being involved in road traffic accidents in areas with heavy traffic, like urban or metropolitan cities, compared to rural areas. These accidents could cause serious injuries (e.g., rupturing internal organs or broken bones) that may result in financial or welfare ramifications (e.g., amputations) or death (16), and may deter owners from allowing outdoor access in high-traffic areas.

Women were less likely to allow uncontrolled outdoor access than men. Studies have shown that women tend to display more positive behaviors and concerns toward animal welfare and animal rights than men (44, 45). Research also suggests female pet owners have a stronger bond with their animals than male owners and this factor increased the likelihood of bringing their pets to the veterinarian for care (46). Furthermore, women have been found to interact with their cats (i.e., physically and verbally) more often than men, supporting the suggestion that women develop higher quality relationships with their cats (47). If women have stronger bonds with their cats, they may be more concerned about the risks associated with outdoor access. Owners with professional degrees (e.g., veterinarians or doctors) also had reduced odds of providing uncontrolled outdoor access, which might be attributable to greater awareness about the risks about this form of outdoor access (e.g., contractible diseases) and about general recommendations for pet care.

### Owner Perspectives Factors

The majority of owner perspectives aligned as predicted, where owners who were concerned about risks to their cat's welfare (e.g., being injured or contracting a disease) had lower odds of providing uncontrolled outdoor access, and owners who agreed with factors related to outdoor access that could enhance their cat's welfare (e.g., reduce risk of behavioral issues) had higher odds of providing uncontrolled outdoor access. These findings are similar to those of another owner-completed survey that found that owners cited various outdoor risks as reasons for keeping their cats indoors (24). Our findings are further supported by previous research with Australian cat owners. One study found that a major barrier to containment was a belief that cats need to wander for mental and physical health (27), and another study found that owners that keep their cats indoors are more likely to believe confinement protects cats from injury (25).

However, not all perspectives aligned. For example, owners who agreed that “outdoor access for cats is problematic because cats hunt small mammals and birds” had higher odds of providing outdoor access. Similarly, other studies have found that beliefs about cats influencing wildlife are either not or only weakly correlated with cat owner behaviors and intentions around keeping cats indoors (26, 27). This misalignment could be due to cognitive dissonance, which is a result of conflict between attitudes and behaviors (48). According to Akpan et al. (49), people's actions do not always align with their beliefs, which could explain how even though owners are aware of the associated risks with outdoor access, they continue to provide access. Alternatively, some owners might have had positive feelings about pest control by cats. For example, Foreman-Worsley et al. (24) found that some owners relied on cats for control of rats and mice on their property. We might have found different results in the current study had we separated reporting for species thought of as pests from songbirds and other “attractive” wildlife. Additionally, there were owners who were not aware of certain risks. For example, only 33.8% of owners agreed that outdoor cats can transmit diseases to humans and animals; however, as Kasbaoui (9) demonstrated, contact with wildlife and other animals can increase transmission of diseases and parasites to cats, and also to other animals via cats. Even when certain risks are widely recognized, owners are still providing uncontrolled access, suggesting a disconnect between knowledge and action. Interestingly, one recent intervention study found that wildlife protection messaging was effective at increasing both motivation to contain and belief that containing was possible in a sample of Australian cat owners, suggesting that further education could be effective for altering cat containment activities (29).

### Enrichment and Home Environment Factors

Other pets living in the cat's home environment increased the odds of having outdoor access. This corresponds to the results of Foreman-Worsley et al. (24) which found that owners cited having a multi-cat household as one of the reasons why they allow their cat outdoor access; open-text responses from owners in this study suggested outside access provided additional space for the cats to get away from each other. Without a safe place to allow escape from unfamiliar or undesired situations (8) the likelihood of having inter-animal aggression or problems between pets is greater. Further research is necessary to explore this relationship.

The provision of interactive enrichment in the home reduced the odds of cats being given uncontrolled outdoor access. This relationship is likely not causal; instead, owners who keep their cats indoors are probably more likely to provide enhanced enrichment to account for the limited and confining indoor environment. The current study found that cats without uncontrolled outdoor access were provided with more enrichment types (e.g., small toys, interactive toys, and scratching areas) than cats with uncontrolled access. Enrichment promotes species-specific behavior, such as chasing, climbing, or biting by imitating prey or natural environments (8). For animals without outdoor access, their indoor environment may be enhanced with enrichment, minimizing boredom and behavioral issues, such as aggression (15). Therefore, while indoor housing can limit physical activity and the ability to perform natural behaviors, providing interactive enrichment can improve their housing quality and promote their well-being.

### Limitations

The majority of the participants reported that they resided in Canada, and most of them resided in Ontario. Furthermore, as is common for online surveys (50), this survey also had more women respondents than men. Due to the disproportionate gender ratio and number of participants from particular regions, it is possible that findings and trends may not generalize broadly to the target population. However, the sample size was relatively large, with reasonable representation from males and different geographical areas. In addition, the regression model accounted for gender through inclusion in analysis as well as geographical clustering through inclusion of participant state/province as a random effect, and while significant, the effect size for state/province was small and significance was likely a result of the large sample size. Additionally, since this survey relied on owner self-reporting, social desirability bias, a bias involving answering based on what is believed to be favorable or are society's norms, may have occurred. Accompanied with owners being unable to accurately recall details, such as average time spent outside, the results of how owners care for their cats and perceive cat welfare may have been skewed.

Since the survey was cross-sectional, capturing prevalence data at one period in time, it is not possible to infer causation or illustrate longitudinal trends. Also, because this was an exploratory and hypothesis-generating study, the high number of variables tested increased the chance of type one errors. The cross-sectional and exploratory nature of this current study, however, highlights areas for future research through controlled studies.

## Conclusions

One fifth of owners in the current survey allowed their cats with uncontrolled outdoor access, and many owners that provided outdoor access failed to implement management strategies that are commonly recommended to protect the welfare of cats and of wildlife, such as microchipping and using a collar with a bell. Several factors that were associated with the provision of uncontrolled outdoor access were identified, including factors related to cat characteristics (e.g., sex, breed, cat age, existing medical conditions, aggression), the home environment (e.g., other animals in the home), owner perspectives (e.g., benefits and risks associated with outdoor access), and owner demographics (e.g., gender, level of education, location, and type of household). The majority of owners were aware of the primary risks and benefits associated with outdoor access, but their attitudes were not the sole factor that influenced the provision of outdoor access. Based on the current results, further research is needed to explore domestic cat needs and the other outdoor alternatives to promote and protect the welfare of owned domestic cats. Outdoor access is a multifactorial decision and future research should explore the impact of outdoor access on cat welfare, the effect of owner-pet bonds on outdoor access and the efficacy of educational programs on owner perspectives toward outdoor access.

## Data Availability Statement

The datasets presented in this article are not readily available because consent was not provided for distribution during ethical approval. However, particular details can be provided upon request. Requests to access the datasets should be directed to Lee Niel at niell@uoguelph.ca.

## Ethics Statement

The studies involving human participants were reviewed and approved by University of Guelph Research Ethics Board-Natural, Physical and Engineering Sciences and The University of British Columbia Behavioral Research Ethics Board. The patients/participants provided their written informed consent to participate in this study.

## Author Contributions

ST and AS organized the database and performed the statistical analysis. ST wrote the first draft of the manuscript. All authors contributed to conception, design of the study, contributed to manuscript revision, read, and approved the submitted version.

## Funding

Publications fees for this article were provided by the Ontario Veterinary College Pet Trust Fund.

## Conflict of Interest

The authors declare that the research was conducted in the absence of any commercial or financial relationships that could be construed as a potential conflict of interest.

## Publisher's Note

All claims expressed in this article are solely those of the authors and do not necessarily represent those of their affiliated organizations, or those of the publisher, the editors and the reviewers. Any product that may be evaluated in this article, or claim that may be made by its manufacturer, is not guaranteed or endorsed by the publisher.
